# Laughter and culture

**DOI:** 10.1098/rstb.2021.0179

**Published:** 2022-11-07

**Authors:** Gregory A. Bryant, Constance M. Bainbridge

**Affiliations:** Department of Communication, Center for Behavior, Evolution, and Culture, University of California, Los Angeles, 2225 Rolfe Hall, Los Angeles, CA 90095, USA

**Keywords:** laughter, cross-cultural, vocal communication, conversation analysis

## Abstract

Like most human non-verbal vocalizations, laughter is produced by speakers of all languages, across all known societies. But despite this obvious fact (or perhaps because of it), there is little comparative research examining the structural and functional similarity of laughter across speakers from different cultures. Here, we describe existing research examining (i) the perception of laughter across disparate cultures, (ii) conversation analysis examining how laughter manifests itself during discourse across different languages, and (iii) computational methods developed for automatically detecting laughter in spoken language databases. Together, these three areas of investigation provide clues regarding universals and cultural variations in laughter production and perception, and offer methodological tools that can be useful for future large-scale cross-cultural studies. We conclude by providing suggestions for areas of research and predictions of what we should expect to discover. Overall, we highlight how important questions regarding human vocal communication across cultures can be addressed through the examination of spontaneous and volitional laughter.

This article is part of the theme issue ‘Cracking the laugh code: laughter through the lens of biology, psychology and neuroscience’.

## 1. Introduction

In research papers or essays on laughter, authors quite often proclaim that laughter is ubiquitous across all cultures. It is common sense to assume that all typically developing people around the world laugh, and additionally we cry, scream, moan, and of course, speak. The various descriptions available regarding human behaviour, whether from social scientists or casual observers of people in action, reveal these facts not through a confirmatory cross-cultural checklist of observed behaviours, but, rather, by not pointing out their absence. The reason we can safely assume that people everywhere laugh is because we have never seen a credible account of a group of people who do not laugh (or more importantly, cannot). Human communication consists of many universal behaviours that vary within and between groups, including in their structures and functions (e.g. [1–6]). Laughter provides us with an excellent case of a communicative behaviour that varies in its manifestations (both acoustically and pragmatically), but appears universally nonetheless. Somewhat surprisingly, there is limited research documenting this variation.

One reason that human non-verbal vocalizations occur universally at some level is because they directly evolved from behaviours in our mammalian ancestors. The underlying brain mechanisms that generate emotional vocalizations, including distress calls, fear screams and threat displays, are shared across virtually all mammals—it is part of what it means to be not only human, but a mammal [7]. This fact makes questions of cultural universality in human vocal communication seem narrower than some might imagine. In the case of laughter, related vocalizations across diverse species reveal a long evolutionary history of vocal play signalling (for a review, see [8]), bringing some perspective to the question of why laughter in humans appears quite similarly across cultures, and offering a framework for understanding how it currently functions. But humans do possess a species-specific capability that complicates the problem for researchers: speech production. Volitional control of speech articulators, operating through direct neural projections between motor cortex and laryngeal musculature, affords the production of complex, rapidly generated phonetic sounds useful for language [7,9]. And the volitional control of articulators allows speakers to imitate other kinds of sounds in our environment [10,11].

One likely target of selection for imitative volitional vocal machinery is evolutionarily conserved, spontaneous emotional vocalizations. That is, there has been positive selection in humans to modulate our voice output to generate volitional versions of our vocal repertoire (e.g. laughs, cries and screams) that we share with most extant mammal species. The benefits of such an ability are fairly clear: if individuals evolve the ability to reliably generate emulations of emotional vocalizations, historically only produced in specific communicative contexts that included constraints of honest signal evolution involving strategic and efficacy costs [12], then agents can gain benefits potentially without paying these costs. That is, the signals can be faked, and consequently dishonest. This additional means of manipulation in vocal signalling introduces complexities for efforts to model its evolutionary dynamics, and is a crucial factor in any theoretical explanation of human vocal communication.

Laughter provides an excellent opportunity for examining the interaction between spontaneous and volitional vocal signalling (e.g. [13,14]). Ultimately, cross-cultural analyses are the only way researchers can uncover the important interactions between universals and cultural variations in vocalizations such as laughter, and most other social behaviour. But what research currently exists in the study of how laughter manifests itself across languages and cultures? There is a surprising lack of work on the topic. And what is the role of humour in understanding how laughter functions across cultures? Here, we describe recent research on (i) the perception of laughter across cultures related to both production mode and social meaning, (ii) conversation analysis (CA) in selected languages, examining the role of laughter during talk, and (iii) computational approaches to automatic laughter detection across various languages. Overall, we argue that laughter occurs in highly similar ways across all documented languages studied to date, and that listeners around the world hear laughter similarly, and are able to make accurate judgements about laughers from very brief exposures (e.g. approx. 1 s), providing initial evidence for cognitive adaptations that extract rich social information from laughter. A theoretical framework is needed that allows researchers to understand differences in communicative behaviours that certainly exist, and recognize them as variations on universal themes with particular constraints.

## 2. Laughter and humour

One important issue when discussing laughter across cultures is the psychology of humour. The interdisciplinary study of laughter has long been closely linked with humour, ranging from early theories of laughter (e.g. [15]), to ethnography (e.g. [16]), to the nature of stimuli used to trigger laughter that is then subjected to acoustic analysis (e.g. [17]). There is little doubt that the complete story of laughter must include humour, but a full review and critique of the literature is beyond the scope of this article.

For our purposes, we assume that events, utterances and ideas can be construed as humorous, and these phenomena are often linked to laughter. We can also safely adopt the premise that people's judgements of what counts as humorous are subject to much cultural variation [18]. Descriptions of the details underlying this variation, and the complicated cognitive underpinnings of humour and social interaction, constitute a proximate analysis of a larger social communication system that includes laughter. The mechanisms by which humour is realized, and how it is effective during discourse, are intimately tied to the ultimate explanations of why people vocalize in the first place. Similarly, detailed analyses of the possible variations in acoustic structure of laughter constitute an additional proximate level of description that can inform ultimate theories regarding purported functions of laughter. In terms of culture, proximate details should be understood as variations on a theme, while understanding the evolutionary dynamics of vocal signalling requires an adaptationist account that applies universally.

The concept of humour has engaged scholars across many disciplines, including but not limited to philosophy, linguistics, psychology, anthropology and even computer science (e.g. [18–22]). There is still no consensus of course, but theorists continue to propose accounts that detail aspects of the environment that afford cognitive effects one might identify as humour. It is worth noting, however, that theories of humour frequently neglect the social interactive context, often in favour of focusing on narrow phenomena such as the structural features of humorous content (e.g. the composition of a joke). Humour should be understood as an emergent property of a socially shared cognitive environment, characterizing multiple people's understanding of that environment and their mutually manifested attempts to engage in it [23,24].

One attempt to integrate different accounts of humour is encryption theory [25], which also provides a framework for understanding the relationship between laughter and humour [26,27]. Briefly, encryption theory proposes that humour is a special case of ostensive communication (i.e. the signalling of an intention to communicate) [28] in which meaning is encrypted (i.e. hidden) such that only receivers with certain information will be able to decrypt it. By relying on pragmatic reasoning required for the comprehension of implied meaning, listeners must derive their understanding of speakers' intentions based on evidence provided in signals (usually, but not always language-based). Success in properly interpreting intentional humour comes with endogenous reward, manifesting subjectively as pleasurable, and often triggering laughter. Proximate rewards are a hallmark of adaptation—the motivational trigger for adaptive action. The motivation to engage socially using encrypted communication promotes the identification and assortment of social partners who share beliefs, worldviews, values and so on. We not only find our social partners using encryption, but help maintain our bonds through it as well, explaining why established friends engage in banter and humour even after social assortment has occurred.

In its simplest manifestation, the encryption–decryption circuit explains both the classic conundrum of the person who fails to get a joke, and the well-known phenomenon of how a joke loses its humour when it must be explained. If an individual does not possess the information necessary to recognize the hidden meaning of a comment, they will fail to get the humour. When a person does have the necessary information, their response often triggers the production of a signal that indicates this shared knowledge, most notably a spontaneous laugh. Of course, an individual can potentially recognize that an encrypted bit of information has been sent without knowing what is encrypted specifically, either to themselves or to another party, and attempt to produce a signal suggesting their understanding. But that signal will be volitionally produced and is at risk of being recognizable to others as faked—a classic scenario for coevolutionary arms race dynamics [13]. This basic social context is the ecology of intentional humour which shapes the evolutionary dynamics of not only laughter, but covert signalling more broadly [29].

The encryption account nicely explains many of humour's most obvious qualities such as its obliqueness, subjectivity and cultural variation. By this view, many theories of humour are potential proximate mechanisms by which encryption does its work. For example, incongruity-resolution theory (e.g. [30]), variants of which are currently among the most popular theories of humour [31], describes a suite of possible encryption mechanisms. Many of these variants involve a multistage process where some incongruity is introduced (e.g. a joke set-up), and then that incongruity is resolved, usually through some unexpected means. The notion dates back centuries (e.g. [15,32]), and is central to more contemporary evolutionary-based accounts of humour as well [20]. The specific content that is introduced into these devices must interface with the knowledge and communication systems of social agents, much of which is culturally shaped.

Encryption theory provides a plausible evolutionary account of not only why people engage using such complex, honest social signalling, but also how laughter plays a role in the signalling circuit. An understanding of laughter across cultures requires an explanation of why people laugh in social settings, and how the content of people's communication triggers it. In other words, cognitive phenomena can be similar across disparate cultures, and cultural factors create variation in content. For instance, one account of humour proposes that benign violations of our expectations will result in humour—potential threats to our state of being or appraisals become funny when they turn out harmless [33]. However, what counts as a violation can often depend on culturally evolved norms—the mechanism could be universal, but the specific content culturally varies. This theory provides another example of a proximate device that ultimately involves indirect communication (i.e. the exact violation is typically not explicitly stated), and is thus subsumed by the more general system of encryption. Overall, an encryption approach predicts that laughter should manifest itself quite similarly across cultures in both form (acoustic structure) and function (pragmatic and social signalling), but what content actually triggers it will be culturally dependent and potentially highly variable. This is due to culture- and language-specific information that can only be understood by individuals immersed in a given socioecological context [34]. Moreover, across different cultures, people vary in how they value humour as a social phenomenon in the first place, and as such can constitute a source of variation in how humour occurs in ordinary interaction [35].

## 3. Perceiving laughter across cultures

If laughter sounds fundamentally similar across different languages and cultures, then we should expect it to be highly recognizable as a distinct vocal behaviour in cross-cultural studies, and potentially judged similarly in terms of social and emotional meaning. But, to date, there is limited empirical work examining how people around the world perceive laughter. The earliest study carefully investigating the cross-cultural perception of non-linguistic vocalizations found that individuals across very different societies (British and Himba) mutually identified laughing as indicative of tickling and amusement [36], despite other positive emotions not being reliably recognized. In follow-up work with the same Namibian population, using similar stimuli and methods, researchers also found that laughter was highly recognized as representing amusement, even though overall this team did not find evidence of widespread universal recognition of other emotion categories [37]; for discussion, see [38]. Even in a free-labelling paradigm, Himba participants mapped American laughter to amusement at a rate far exceeding any other emotion category, despite the many reasons we should expect high cultural variability using such a method. Moreover, they judged laughter as highly positive in valence and arousal.

More recently, two large-scale studies investigated how listeners across multiple disparate cultures judged recordings of laughing. One study [39] examined the perception of conversational colaughter, defined as the simultaneous production (onsets within 1 s) in two interacting speakers of non-verbal laughter bursts. Colaughter samples extracted from spontaneous conversations were played to listeners (*N* = 966) from 24 different societies, ranging from WEIRD^1^ college students to small-scale hunter–gatherers. The colaughter originated from conversations obtained between established friends or newly acquainted strangers, in all gender combinations. The extracted 48 colaugh stimuli averaged approximately 1 s in duration. The task was simple: judges were asked to listen to the colaughter and then report (i) whether they believed the presented pair of speakers were friends or strangers, and (ii) how much did they think the speakers liked one another.

Results across cultures were strikingly similar. Overall, judges everywhere were able to correctly classify friend and stranger pairs significantly better than chance, ranging from 53 to 67% accuracy. Listeners additionally agreed widely on how much the speakers liked one another, with average ratings in all cultures showing the expected difference of friends liking one another more than strangers. An acoustic model was created to identify structural features of the individual laugh samples that predicted people's judgement of friendship in the dyads. Laughs with shorter duration, less regular pitch and intensity cycles, and less variation in pitch cycle regularity were more likely to be judged as between friends. These acoustic qualities are all associated with speaker arousal, suggesting that friendly colaughter likely reveals an affective correlate of familiarity in ordinary conversation.

One unexpected and pronounced finding was that across all cultures—without exception—judges identified female pairs of friends most accurately across the six categories (friends and strangers across M–M, F–F and M–F combinations). The accuracy difference is partially due to a culturally universal bias to overestimate the likelihood of reporting that a pair was friends when they were women (i.e. high false alarms). But the individual laughs making up the female pairs were also judged as sounding relatively more aroused, and with more positive valence, than individual laughs from other dyad types. Finally, across all cultures, pairs of female friends were judged to have liked each other more. Together, these results speak to (i) the widespread importance of speaker affect in how laughter manifests itself in socially dependent ways, and (ii) how rapidly people around the world can make accurate, socially relevant judgements, in this case within 1 s.

A follow-up cross-cultural study examined whether listeners across a similarly diverse (and overlapping) set of 21 societies could distinguish between spontaneous and volitional laughter [42]. For this work, and the study from which the stimuli were created [13], spontaneous (real) laughter was defined as conversational laughter obtained between familiar speakers, and all exemplars were between female friends. Volitional (fake) laughter was defined as laughter produced on command in a laboratory context with no other instruction other than to ‘now laugh,' and this sample was also composed of all women. These definitions were employed for practical reasons and, of course, were not perfect. Namely, the actual distinction between spontaneous and volitional laughter is based on which vocal production system is physically invoked, only confirmable through brain imaging analyses. But a conflation of categories in some stimuli works against the prediction that listeners should be able to distinguish the laughter types.

In Bryant *et al.* [42], a set of 36 laughs (18 spontaneous) were presented to listeners (*N* = 884) worldwide and they were asked to judge whether they thought the laughter was ‘real' or ‘fake.' As expected, participants everywhere, on average, were able to distinguish the laugh types with above-chance performance (56–69%). Moreover, arousal-linked acoustic features, including higher intensity variability, higher fundamental frequency (*f*_o_), and lower harmonics-to-noise-ratio variability, were associated with listeners judging laughs as ‘real'. Ultimately, it is likely that spontaneous laughter is more common between friends than strangers, and arousal is a basic low-level dimension that distinguishes it from volitional laughter more common between strangers. This fundamental connection between affect and sociality is highly recognizable universally.

One interesting cultural difference emerged in the study of spontaneous and volitional laughter [42]. Participants from small-scale societies, defined roughly as people living in small villages with low market integration, had a slight bias to report laughs as volitional, or ‘fake' as they were asked. This bias caused them to be more accurate for this category (at the expense of accuracy for identifying spontaneous laughter). It is impossible to know what exactly caused this criterion shift. One possibility is that societies composed of relatively smaller groups of recurrent players might result in individuals sensitized to the volitional nature of social pleasantries that better characterize larger societies where a much higher proportion of social interactions are between people who have never met and could easily not have a repeated encounter. Put simply: they think American college students sound fake. Because of potential greater costs of errors in more close-knit communities, heightened sensitivity to emotion and intention detection could be adaptive in small-scale societies to a greater extent than in larger, industrialized societies where one-shot interactions with strangers are commonplace and generally benign.

Another relevant aspect of this finding is the distinct possibility that several of the laughs included in the spontaneous condition were in fact volitional. The laughter was defined based on having occurred between friends in conversation, but many laughs between familiar speakers (in fact, perhaps a majority) are generated by the speech production system (making them volitional), and serve a variety of pragmatic and procedural functions. Most so-called ‘fake' laughs are probably not deceptive, and in fact serve as reliable signals for a variety of discourse functions. But they are not emotionally triggered in the same way as spontaneous laughs. By this logic, small-scale participants were likely more accurate overall. In any case, the ease with which individuals worldwide were able to engage in the task and understand what was being asked of them, despite many having no experience with social science research, speaks to the universal nature of laughter—people everywhere get it.

Based on the idea that spontaneous and volitional laughter are generated distinctly by our two vocal production systems (i.e. phylogenetically widespread vocal emotions and species-specific speech), fairly clear predictions emerge for several research areas of laughter, including speaker identity [43], in-group/out-group laughter distinctions [44], and laughter acoustics across speakers of different languages [45]. If volitional laughter is generated by the speech production system, then linguistically variable speech features are likely often incorporated into a person's volitional laughter and consequently identifiable to listeners. For example, phonetic properties of vowel sounds that differ across languages could manifest themselves in volitional laughter. But studies have described incredible acoustic variability in laughter even within speakers of English, revealing, for example, that laughs range in structure from very short, quiet, broadband bursts of air, to extended sequences of loud, tonal bouts, with many variants in between [17]. Spontaneous laughter, conversely, does not contain speech features and thus should be indistinguishable across highly disparate languages and cultures. No large-scale study has examined this, but results from a couple of smaller studies suggest that it might be complicated.

Recently, Kamiloglu *et al*. [44] found that group membership (Dutch and Japanese) could be reliably identified by listeners hearing both spontaneous and volitional laughter. Overall, the spontaneous laughter was judged as more positive-sounding by all listeners, and Dutch listeners (but not Japanese listeners) thought laughter from their in-group was more positive. As described earlier, knowing what category a laugh belongs to can only be confirmed with certainty by examining cortical activity during production. One possibility in this study is that a majority of the laughs were in fact volitional, but varied in portrayed arousal (causing some to be judged as spontaneous), and as such revealed group identity. The spontaneous laughter was recorded while participants watched a self-selected funny video, but laboratory conditions can easily hinder spontaneous performances. An earlier study [46] found that Dutch listeners could not discern in-group from out-group vocalizers from a set of laughs including speakers of six languages (Dutch, English, French, US American, Japanese and Namibian). But spontaneity was not tightly controlled in the stimulus set, and the task was rather difficult, with listeners needing to choose from six categories. More research is certainly needed to explore this issue.

The literature on laughter perception across cultures reveals robust universals in basic identification of laughter as a signal of amusement, as well as widely shared intuitions about the relationships between people laughing together, and the social meanings of individual laughs as indexed by the distinction between spontaneous and volitional laughter. It is not surprising that such an ancient vocalization, shaped by form–function principles across mammal species, is widely recognizable across all people living today [47]. But laughter is a socially complex vocalization in the human repertoire, adapted to social interactions that include language, gestures, facial expressions and mindreading. We are social agents engaging using ostensive communication strategies that are integrally tied to our sophisticated social cognition [28,48]. Many of the practices involved in everyday conversation are shaped by culture, including of course language itself, and we should expect many, if not most, behavioural phenomena operating in this space to be affected by culturally specific practices. There is unfortunately a serious lack of research examining any non-verbal vocalization in a comparative way across a large number of languages and cultures, but a variety of smaller studies in conversation analysis and affective computing provide some clues.

Thus far, it appears that while laughter can manifest itself in an amazing variety of ways, there do not appear to be any deep systematic cultural differences in how it operates. Below, we describe some of this work, illustrating the similarities and variation in not only how laughter appears in different conversational language users, but also how it can be automatically detected in speech streams. These research areas offer useful tools that researchers can use moving forward.

## 4. Conversation analysis and sociolinguistics

CA is an approach to the study of language and social interaction, involving fine-grained, often qualitative analyses of the sequential organization of interactive behaviour, typically involving language [49–51]. One beauty of CA is the deep appreciation for spontaneous discourse, and the description of all the behavioural signals that co-occur with talk-in-interaction, including laughter. But few studies in CA have examined laughter comparatively across languages, and instead studies usually focus on a single language. CA researchers have described laughter behaviour in many Indo-European language speakers, in addition to Chinese, Japanese, Korean and others. In every case, the described phenomena are highly recognizable to any monolingual English speaker. For example, people often smile in response to laughter, cede the floor when speaking just after a laugh, terminate a topic when laughing together, laugh when a pause lasts more than half a second, manage disagreements or colaugh during the mutual recognition of a ‘laughable' (i.e. a laugh trigger, usually referred to in conversation directly or indirectly) or to mark a ‘play frame' more generally [52–58]. Overall, laughter seems to function universally as an organizer of talk, and as a complex pragmatic signal fulfilling a wide array of discourse functions.

The study of pragmatic function in CA takes various forms. Analyses typically focus on conversational actions and organization, such as how people manage turn-taking and mechanisms of repair, but many scholars have investigated higher-level phenomena such as speech acts (e.g. promises and apologies) (e.g. [59]) and indirectness (e.g. implicature) [60]. Pragmatic functions in spontaneous interaction can be demonstrated by example, or coded and quantified, with the latter becoming increasingly common. Since much of this scholarship has traditionally been on single languages, concerns regarding metalinguistic knowledge and cultural equivalence have often been neglected. But these components of social interaction are needed for the proper cross-cultural investigation of pragmatic function [61].

One pattern we see repeatedly in cross-cultural analyses of communicative behaviour is that a single pragmatic function can be realized through multiple vocal or other behaviours, which can be subject to cultural variation. Studies of infant-directed (ID) speech research provide a nice illustration. There are a variety of choices for a caregiver when attempting to verbally encourage some behaviour in a young child (i.e. approval utterances). Local social conventions could shape caregivers' behaviour to slow down precipitously, for example, or perhaps to increase the pitch, or even do both [62]. In some societies, speaking directly to infants is relatively less common (e.g. [63]), so vocal tactics used by caregivers in one place might be accomplished through non-vocal means in another. But, in the end, these strategies can be comparably effective. Thus, an analysis showing a difference across cultural groups on a specific altered vocal dimension in ID speech is not evidence against universality in ID speech more generally, but rather a demonstration of variation within the domain of a universally occurring phenomenon [5,38]. CA research also provides examples of speakers across disparate cultures accomplishing various discourse functions using different strategies, but the function itself is intact universally [61].

Laughter is likely to follow this type of cultural patterning as well, with a broad suite of universal pragmatic functions being fulfilled variably by laughter and other interactive behaviours. While still relatively uncommon, some researchers have examined conversational laughter comparatively across languages using CA methods, or other similar coding techniques. Mazzocconi *et al*. [64] offered a nice review of various taxonomies theorists have developed for laughter and pointed out rightly that many attempts to categorize laughter types and functions confuse levels of analysis, conflating, for example, phonetic categories with pragmatic ones. These authors presented a new taxonomy that avoids this pitfall. They proposed that laughter constitutes an affective force (with some core essence) that is contextually appraised in relation to any number of laughables, including many containing incongruities of one sort or another, and others not. For instance, a social incongruity might be laughing from embarrassment and a pragmatic incongruity might be an ironic utterance, but an affiliative laugh might just signal closeness. These categories are identified independently from the underlying basis of the laughter production, such as whether laughs are spontaneous or volitional, or what emotions might be gleaned from them when presented without context in perceptual tasks.

Using this multilayered framework, Mazzocconi *et al*., [64] examined an audiovisual corpus of task dialogue (DUEL) in French and Mandarin Chinese (9 dyads total), and variable conversations in British English from the audio-only BNC corpus (21 dyads). Laughter, laughables and functions were coded, and some differences emerged. For example, Chinese speakers laughed relatively more frequently at social incongruities than pragmatic incongruities, and also had relatively lower arousal laughter. Additionally, laughter in Chinese speakers tended to be more often speech-laughs, which is laughter that co-occurs with speech. Proportions of laughs across social and pragmatic categories were similar across languages, however. Because of the somewhat small sample sizes, generalizations regarding observed differences are difficult to make, and the differing contexts of the conversations used could potentially explain all the variation. But the analysis did reveal that features of the laughs, such as the arousal indicated in the speakers and the relation of the laugh positions to the laughables, did not predict the functions, indicating notable variation in how laughter occurs in conversation across languages. The lack of substantive differences across these three languages supports the idea that language and culture-based variations might have negligible effects on the ways laughter operates in discourse.

Dingemanse & Floyd [61] described the natural control method in CA for comparative analysis, such as identifying equivalent turn-taking structures for assessing exchange times [6] or looking at similarly structured turn contexts for analysing repair efforts [2]. Cultural differences in conversational laughter might be more evident at the turn level between conversation partners, rather than the utterance level, where evidence suggests universal trends [65]. For instance, Gavioli [66] found that during bookshop service encounters in England and Italy, similarities and differences in the assistants' uses of laughter were observed. While both used laughter in the context of a dispreferred response (e.g. a desired book being unavailable), in the English corpus, laughter was turn-initial and prefacing an excuse or other account. In the Italian corpus, the laughter occurred at the end of their conversational turn, leaving open discussion for resolution of the situation. Recently, Ludusan & Wagner [67] examined how conversationalists across different languages laughed in coordinated ways, and found similar patterns of entrainment across French, German and Mandarin Chinese speakers. Speakers in all languages concentrated their laughter around turns, and also tended to converge in the temporal structure of their laughter over time during a conversation. While overall there is limited work investigating how laughter occurs across different languages, much of the variability in how laughter appears during talk—whether in relation to pragmatic functions like those studied by Gavioli [66], or more mechanistic analyses [67]—could be limited through tighter controls on the many relevant contextual variables at play during conversation.

## 5. Computational approaches

One burgeoning area of laughter research explores the distinction between laughter and speech, and how we might develop machines to automatically decompose natural speech. Machine learning techniques are frequently used in such analyses for detecting laughter. Most generally, machine learning is the use of algorithms to perform tasks, such as classification, without being programmed explicitly to do so. Algorithms are typically ‘trained' on a set of tokens, and then, based on any detectable information structure in that set, can be used to perform classification on novel databases. An interesting, though at times frustrating, aspect of the technique is that it can be impossible to know exactly how the algorithms learn the classification. Additionally, the techniques are often developed by industry to solve computational problems in automatic recognition systems or develop human–computer interfaces, and not for basic research on human communication.

One such technique is the use of Support Vector Machines (SVM) which algorithmically learn to assign labels for classifying objects, represented as points within a high-dimensional space, and divided based on maximum-margin separating classes (for a comprehensive explanation of SVM, see [68]). Another approach, Gaussian Mixture Models (GMM), are frequently used for modelling the features of vocal-tract systems to understand spectral features of speech and laughter. GMM include the clustering of data points with probabilistic considerations, rather than assigning points to categories based only on the closest cluster's centre, and as such can provide more nuanced classification of vocalization information. Truong & van Leeuwen [69] developed models fusing SVM and GMM using the English ICSI corpus that were able to perform well with Dutch recordings from the CGN corpus. But owing to differences in recording contexts, differences in model performance across the corpora are difficult to interpret. These studies point to the universal structure in laughter—machines trained on laughter phenomena in one language can often perform equally well on different languages.

The features considered when employing machine learning techniques may also matter. Neuberger & Beke [70] found a combination of SVM and GMM techniques increased discriminative power for detecting laughter in Hungarian spontaneous speech samples. Of the feature sets tested in their study, Mel-Frequency Cepstral Coefficients (MFCCs) gave the best results. MFCCs use filters that closely resemble the variability of the human ear, and can be used to characterize speech signals [71]. While MFCC may provide power in laugh detection, other features may provide greater accuracy. In a comparison between MFCC, Perceptual Linear Prediction (PLP) and raw mel-scale filter bank energies (FBANK) in detecting laughter, FBANK performed the best for both Hungarian (studio-recorded) and English (telephone) recordings [72]. These mel-scale filter bank energies consider the breakdown of sound input into different frequencies that are perceived as equally spaced apart. Despite the corpora used in this study differing dramatically in audio quality owing to their recording contexts, some language-independent aspects of laughter were still detectable. Future work will benefit from exploring a highly controlled set of cross-linguistic stimuli to parse out the details of which features best reveal similarities versus cultural differences of laughter.

Different machine learning strategies vary in their success rates for classification of laughter types. A study on detecting emotions in Filipino laughter found that Multilayer Perceptron (MLP) yielded a higher correct classification rate (at 44%) compared with using SVM (18%) [73]. MLP considers the weights within a network to select features, and may be better suited for audio datasets, while SVM may perform better for video in cases where multimodal information is available [74]. SVM has also been used to classify laughter as polite or mirthful for a Japanese, Chinese and English dataset with at least 85% accuracy [75]. Not surprisingly, the use of multimodal information is likely to become the gold standard for accurate detection and classification of laughter. For example, the combination of smile detection in images with acoustic detection via GMM revealed increased accuracy in detecting laughter, with an end result of a 70% recall and precision rate from natural conversational videos in Japanese, English and Chinese [76]. But if only one modality is available, the sound of a laugh may provide more accuracy than video of the face [77].

Overall, these techniques could be used for large-scale analyses involving many languages. Computational approaches to laughter detection in natural speech reveal tractable distinctions between laughing and speaking, and hint at stability in this difference that transcends language groups. But, to date, no carefully designed comparative study has explored how machine learning might be able to accomplish accurate detection across speakers from different societies.

## 6. Conclusion

Laughter is clearly a human universal, but surprisingly little research is available that confirms the extent to which the properties of laughter are consistent across speakers from different cultural groups. Research has revealed that listeners across a wide array of societies perceive laughter similarly, including the findings that judges worldwide can detect friends versus strangers at levels well above chance, and can also distinguish spontaneous laughter between friends from volitional laughter produced on command. It is unclear at this point whether listeners can identify individuals as in-group versus out-group based only on laughter, and how this relates to whether laughter is spontaneous or volitional.

CA research has described the many ways laughter occurs in ordinary discourse, but only limited work has systematically compared the ways laughter operates across speakers of different languages. The comparative work that does exist currently suggests that the similarities across different languages are notable, but results suggesting any differences are always difficult to interpret owing to a variety of factors, including reliance on different corpora that have confounding differences between them, small samples of speakers, and failures to properly match sample participants. Nevertheless, recent efforts at recognizing the importance of the metalinguistic knowledge of interlocutors, ethnographic details of communicative contexts, and cultural equivalency in measurements hold great promise for CA techniques to reveal cultural universals and variations in how laughter functions in discourse.

Clearly, the most basic need in this research area is a large-scale study that potentially includes all of the methodological approaches described here. High-quality recordings of laughter should be obtained carefully using a standardized method across many societies. Laughter should be extracted from natural conversations between controlled dyads and perhaps larger groups, with other baseline vocal recordings generated from the same individuals (e.g. monophthong vowels, standardized sentences and volitional emotional vocalizations). Ideally, spontaneous laughter from real interactions can be compared with volitional laughter by the same speakers, both acoustically and perceptually. Conversation recordings can be transcribed and subjected to CA methods so laughter can be coded in a way that affords analyses of context and pragmatic function (e.g. [64]). Machine learning-assisted acoustic analysis of a well-controlled database of laughs, produced in highly similar contexts, can explore to what extent the language of a speaker predicts laughter structural features, and whether production mode (i.e. spontaneous or volitional) matters. Finally, perception experiments on such a controlled corpus of laughs can begin to answer an amazing variety of questions related to cross-cultural recognition of specific aspects of laughter that currently cannot be tested.

Given the limited data thus far, it is not completely clear what we might expect from such a thorough examination, but there are some reasonably obvious effects to expect. Our prediction is that volitional laughter produced by the speech system should be more variable across different language speakers than spontaneous laughter produced by the evolutionarily conserved vocal emotion system. Moreover, the degree to which listeners are able to distinguish in-group versus out-group laughter might vary as a function of the cultural distance between the target speaker and the listener [78]. A clear result demonstrating that listeners can distinguish in-group from out-group in spontaneous laughter will present challenges for our current understanding of dual-system vocal production dynamics [10].

Another prediction concerns the pragmatic and social functions of laughter. We might expect high intra- and inter-cultural variability in how laughter occurs in discourse, both in relation to laughables, and also related to people's own speech and others' speech. We should see anomalous uses of laughter in addition to highly regularized patterns, but we should not see systematic uses of laughter in a single culture that do not translate to some kind of discourse function in another language or culture. In other words, any function that laughter fulfils in a given language will represent a function that is fulfilled in every other language, either by laughter or by another strategy. But this is less a claim about laughter *per se*, and instead a claim about discourse across cultures. This is not to say that some cultures have not evolved idiosyncratic communicative phenomena that are unique to that place (they surely have), but if so, they will be immediately understandable to individuals from any other place when properly contextualized.

Finally, another issue that needs more attention, and can be effectively addressed through cross-cultural examination, is the extent to which laughter acoustic features differentiate across interactive functions—are there different laugh ‘types'? Attempts to demonstrate that laughter can be distinguished across social functions in English speakers suggest there could be some distinctions that could reveal themselves widely (e.g. [79]), but other work suggests that context does a lot of the work and that laughter acoustic features are often quite ambiguous [80]. The only currently robust categories of laughter are spontaneous and volitional types, rooted in the distinct vocal production systems underlying them. One possibility is that simple perceptible dimensions such as arousal and valence, which have many known acoustic correlates, can help judges make better-than-chance categorizations of laughter in various experimental paradigms. But systematically linking the acoustic features to more specific functional categories will prove to be untenable.

Laughter stands as one of several non-verbal vocal expressions, along with crying, screaming and others, that are beginning to be extensively explored across cultures, and will help us understand the highly complex communicative behaviours characteristic of our species. New technological developments have provided researchers with many new tools and techniques for conducting large-scale studies, and it is time to begin definitively answering questions concerning the universals and cultural variations underlying human behaviour, including, quite importantly, the nature of laughter.

## Data Availability

This article has no additional data.

## Endnote

1 WEIRD (Western, educated, industrialized, rich, democratic) is a term originally coined by Henrich, Heine & Norenzayan [40]. In the social sciences, researchers have relied on a rather narrow demographic of college-aged students primarily from the global North. For decades, theorists have generalized findings from this research to humans at large, resulting in many misconceptions regarding cultural universals and variation. As the acronym implies, there is accumulating evidence that rather than being anywhere near a typical human demographic, WEIRD individuals are in fact extreme on many relevant dimensions for research on cognition and behaviour. For a recent discussion of ramifications, see [41].
